# Supplementary material for: Effects of a Novel Infant Formula on Weight Gain, Body Composition, Safety and Tolerability to Infants: The INNOVA 2020 Study
Source: Nutrients. 2022 Dec 28;15(1):147. doi: 10.3390/nu15010147 (PMC9823847; doi:10.3390/nu15010147)
Supplement: Supplementary file 1 [file nutrients-15-00147-s001.zip › nutrients-2090068-Supplementary.pdf]

Article

Effects of a Novel Infant Formula on Weight Gain, Body Composition, Safety and Tolerability of Infants: The INNOVA 2020 Study

Julio Plaza-Diaz[1,2,3,#], Francisco Javier Ruiz-Ojeda[1,2,4,5,#], Javier Morales[6], Ana Isabel Cristina de la Torre[7], Antonio García-García[8,9], Carlos Nuñez de Prado[10], Cristóbal Coronel-Rodríguez[11], Cyntia Crespo[11], Eduardo Ortega[12], Esther Marín-Pérez[13], Fernando Ferreira[14], Gema García-Ron[15], Ignacio Galicia[8], María Teresa Santos García-Cuéllar[8], Marcos Moroto[8], Paola Ruiz[16], Raquel Martín[17], Susana Viver-Gómez[18] and Angel Gil[1,2,5,19,*]

[1]Department of Biochemistry and Molecular Biology II, School of Pharmacy, University of Granada, 18071, Granada, Spain
[2]Instituto de Investigación Biosanitaria IBS.GRANADA, Complejo Hospitalario Universitario de Granada, Granada 18014, Spain
[3]Children's Hospital of Eastern Ontario Research Institute, Ottawa, ON K1H 8L1, Canada.
[4]RG Adipocytes and Metabolism, Institute for Diabetes and Obesity, Helmholtz Diabetes Center at Helmholtz Center Munich, Neuherberg, 85764 Munich, Germany.
[5]Institute of Nutrition and Food Technology "José Mataix", Centre of Biomedical Research, University of Granada, Avda. del Conocimiento s/n. 18016 Armilla, Granada, Spain
[6]Product Development Department, Alter Farmacia SA, 28880 Madrid, Spain.
[7]CS Presentación sabio. C/Alonso Cano 8, 28933 Móstoles, Madrid
[8]Instituto Fundación Teófilo Hernando (IFTH). Parque científico de Madrid. UAM.. C/ Faraday 7. Edificio CLAID. 28049. Madrid.
[9]Departamento de Farmacología, Facultad de Medicina, Universidad Autónoma de Madrid, Madrid, Spain
[10]Consulta Privada Carlos Núñez, C/Santiago Apóstol 10, 28220 Majadahonda, Madrid, Spain
[11]Centro de Salud Amante Laffón, Distrito de Atención Primaria Sevilla, Servicio Andaluz de Salud.
[12]CAP Nova Lloreda, Av. De Catalunya 62-64, 08917 Badalona, Barcelona.
[13]CS Parque Loranca, C/ de la Alegría 2, 28942 Fuenlabrada, Madrid
[14]Consulta Externa Hospital Privado Santa Ángela de la Cruz, Av. De Jerez 59, 41013, Sevilla.
[15]CS La Rivota. C/ de las Palmeras s/n, 28922 Alcorcón, Madrid.
[16]CS Las Américas, Av. De América 6, 28983 Parla, Madrid.
[17]CS Doctor Luengo Rodríguez, C/ Nueva York 16, 28938 Móstoles, Madrid.
[18]CS Valle de la Oliva. C/ Enrique Granados 2, 28222 Majadahonda, Madrid.
[19]CIBEROBN (CIBER Physiopathology of Obesity and Nutrition), Instituto de Salud Carlos III, 28029 Madrid, Spain

* Correspondence: agil@ugr.es Tel.: +34 695466922 Department of Biochemistry and Molecular Biology II, School of Pharmacy, University of Granada, Campus de Cartuja s/n, 18071, Granada, Spain

[#] Equally contributed

Table S1. Body mass index, weight, and height percentiles.

Body mass index percentiles				P-value		
**Visits**	**BFD (N=58)**	**STD (N=65)**	**INN (N=62)**	**Formula**	**Visit**	**Formula x visit**
**Visit 1**	0.45 ± 0.26	0.42 ± 0.24	0.47 ± 0.25			
**Visit 2**	0.45 ± 0.25	0.43 ± 0.25	0.41 ± 0.26			
**Visit 3**	0.40 ± 0.24	0.44 ± 0.29	0.50 ± 0.29	0.504	0.009	0.006
**Visit 4**	0.43 ± 0.26	0.47 ± 0.31	0.55 ± 0.31			
**Visit 5**	0.51 ± 0.28	0.57 ± 0.31	0.55 ± 0.31			
Height percentiles				**P-value**		
**Visits**	**BFD (N=58)**	**STD (N=65)**	**INN (N=62)**	**Formula**	**Visit**	**Formula x visit**
**Visit 1**	0.54 ± 0.30	0.43 ± 0.30	0.48 ± 0.26			
**Visit 2**	0.58 ± 0.33	0.52 ± 0.30	0.60 ± 0.27			
**Visit 3**	0.52 ± 0.31	0.55 ± 0.30	0.59 ± 0.27	0.433	0.011	<0.001
**Visit 4**	0.47 ± 0.30	0.52 ± 0.31	0.56 ± 0.26			
**Visit 5**	0.53 ± 0.31	0.58 ± 0.30	0.63 ± 0.27			
Weight percentiles				**P-value**		
**Visits**	**BFD (N=58)**	**STD (N=65)**	**INN (N=62)**	**Formula**	**Visit**	**Formula x visit**
**Visit 1**	0.49 ± 0.27	0.40 ± 0.27	0.47 ± 0.25			
**Visit 2**	0.51 ± 0.26	0.45 ± 0.27	0.49 ± 0.25			
**Visit 3**	0.43 ± 0.26	0.47 ± 0.30	0.54 ± 0.26	0.368	<0.001	<0.001
**Visit 4**	0.42 ± 0.25	0.48 ± 0.32	0.56 ± 0.28			
**Visit 5**	0.53 ± 0.26	0.59 ± 0.32	0.61 ± 0.29			

Data are expressed as mean and standard deviation

Table S2. Stool Characteristics.

	Visit 1			Visit 2			Visit 3			Visit 4			Visit 5		
Stool consistency	BFD	STD	INN	BFD	STD	INN	BFD	STD	INN	BFD	STD	INN	BFD	STD	INN
Soft	3 (4.3%)	22 (31.4%)	12 (17.1%)	5 (7.7%)	17 (25.8%)	12 (18.8%)	8 (13.1%)	16 (24.2%)	13 (20.6%)	3 (5.2%)	16 (24.2%)	12 (19.0%)	11 (19.0%)	13 (20.0%)	10 (16.1%)
Hard	5 (7.1%)	6 (8.6%)	11 (15.7%)	0 (0.0%)	1 (1.5%)	3 (4.7%)	0 (0.0%)	0 (0.0%)	1 (1.6%)	1 (1.7%)	4 (6.1%)	0 (0.0%)	4 (6.9%)	7 (10.8%)	5 (8.1%)
Liquid	27 (38.6%)	4 (5.7%)	7 (10.0%)	24 (36.9%)	0 (0.0%)	0 (0.0%)	13 (21.3%)	0 (0.0%)	1 (1.6%)	9 (15.5%)	1 (1.5%)	4 (6.3%)	0 (0.0%)	0 (0.0%)	0 (0.0%)
Normal	0 (0.0%)	3 (4.3%)	2 (2.9%)	0 (0.0%)	4 (6.1%)	1 (1.6%)	2 (3.3%)	7 (10.6%)	3 (4.8%)	2 (3.4%)	8 (12.1%)	2 (3.2%)	7 (12.1%)	9 (13.8%)	16 (25.8%)
Pasty	8 (11.4%)	31 (44.3%)	34 (48.6%)	13 (20.0%)	40 (60.6%)	45 (70.3%)	13 (21.3%)	38 (57.6%)	38 (60.3%)	23 (39.7%)	33 (50.0%)	42 (66.7%)	36 (62.1%)	36 (55.4%)	31 (50.0%)
Semiliquid	27 (38.6%)	4 (5.7%)	4 (5.7%)	23 (35.4%)	4 (6.1%)	3 (4.7%)	25 (41.0%)	5 (7.6%)	7 (11.1%)	20 (34.5%)	4 (6.1%)	3 (4.8%)	0 (0.0%)	0 (0.0%)	0 (0.0%)

Data are expressed as counts and percentages

Table S3. Infant's behavior

Visit		BFD	STD	INN
1	N	70	70	70
	Altered mood	27 (38.6%)	29 (41.4%)	22 (31.4%)
	Good mood	43 (61.4%)	41 (58.6%)	48 (68.6%)
2	N	65	66	64
	Altered mood	22 (33.8%)	18 (27.3%)	18 (28.1%)
	Good mood	43 (66.2%)	48 (72.7%)	46 (71.9%)
3	N	61	66	63
	Altered mood	16 (26.2%)	9 (13.6%)	13 (20.6%)
	Good mood	45 (73.8%)	57 (86.4%)	50 (79.4%)
4	N	58	66	63
	Altered mood	13 (22.4%)	13 (19.7%)	7 (11.1%)
	Good mood	45 (77.6%)	53 (80.3%)	56 (88.9%)
5	N	58	65	62
	Altered mood	10 (17.2%)	4 (6.2%)	6 (9.7%)
	Good mood	48 (82.8%)	61 (93.8%)	56 (90.3%)
Total	N	312	333	322
	Altered mood	88 (28.2%)	73 (21.9%)	66 (20.5%)
	Good mood	224 (71.8%)	260 (78.1%)	256 (79.5%)

Data are expressed as counts and percentages

Table S4. Tolerability and overall assessment by parents or guardians across the study

Tolerability	BFD (N=65)	STD (N=66)	INN (N=64)	Overall assessment by parents or guardians	BFD (N=65)	STD (N=66)	INN (N=64)
Visit 2				Visit 2			
Adequate	5 (7.7%)	4 (6.1%)	1 (1.6%)	Acceptable	1 (1.5%)	3 (4.6%)	2 (3.1%)
Good	50 (76.9%)	48 (72.7%)	50 (78.1%)	Good	54 (83.1%)	40 (61.5%)	47 (73.4%)
Very good	7 (10.8%)	13 (19.7%)	13 (20.3%)	Very good	9 (13.8%)	16 (24.6%)	14 (21.9%)
Data not available	2 (3.1%)	0 (0.0%)	0 (0.0%)	Satisfactory	0 (0.0%)	5 (7.7%)	1 (1.6%)
Not satisfactory	1 (1.5%)	1 (1.5%)	0 (0.0%)	Not satisfactory	1 (1.5%)	1 (1.5%)	0 (0.0%)
Visit 3				Visit 3			
Tolerability	BFD (N=65)	STD (N=66)	INN (N=64)	Overall assessment by parents or guardians	BFD (N=65)	STD (N=66)	INN (N=64)
Adequate	3 (4.9%)	5 (7.6%)	2 (3.2%)	Acceptable	6 (9.8%)	9 (13.6%)	2 (3.2%)
Good	38 (62.3%)	47 (71.2%)	37 (58.7%)	Good	37 (60.7%)	40 (60.6%)	40 (63.5%)
Very good	14 (23.0%)	13 (19.7%)	23 (36.5%)	Very good	15 (24.6%)	13 (19.7%)	19 (30.2%)
Data not available	3 (4.9%)	0 (0.0%)	1 (1.6%)	Satisfactory	0 (0.0%)	3 (4.5%)	2 (3.2%)
Not satisfactory	3 (4.9%)	1 (1.5%)	0 (0.0%)	Not satisfactory	3 (4.9%)	1 (1.5%)	0 (0.0%)
Visit 4				Visit 4			
Tolerability	BFD (N=58)	STD (N=66)	INN (N=63)	Overall assessment by parents or guardians	BFD (N=58)	STD (N=66)	INN (N=63)
Adequate	2 (3.4%)	8 (12.1%)	1 (1.6%)	Acceptable	2 (3.4%)	9 (13.6%)	1 (1.6%)
Good	39 (67.2%)	44 (66.7%)	38 (60.3%)	Good	40 (69.0%)	44 (66.7%)	41 (65.1%)
Very good	14 (24.1%)	13 (19.7%)	23 (36.5%)	Very good	14 (24.1%)	9 (13.6%)	17 (27.0%)
Data not available	3 (5.2%)	0 (0.0%)	0 (0.0%)	Satisfactory	2 (3.4%)	4 (6.1%)	3 (4.8%)
Not satisfactory	0 (0.0%)	1 (1.5%)	1 (1.6%)	Not satisfactory	0 (0.0%)	0 (0.0%)	1 (1.6%)
Visit 5				Visit 5			

Tolerability	BFD (N=58)	STD (N=65)	INN (N=62)	Overall assessment by parents or guardians	BFD (N=58)	STD (N=65)	INN (N=62)
Adequate	2 (3.4%)	5 (7.7%)	0 (0.0%)	Acceptable	2 (3.4%)	9 (13.6%)	1 (1.6%)
Good	33 (56.9%)	34 (52.3%)	40 (64.5%)	Good	40 (69.0%)	44 (66.7%)	41 (65.1%)
Very good	18 (31.0%)	24 (36.9%)	21 (33.9%)	Very good	14 (24.1%)	9 (13.6%)	17 (27.0%)
Data not available	4 (6.9%)	0 (0.0%)	0 (0.0%)	Satisfactory	2 (3.4%)	4 (6.1%)	3 (4.8%)
Not satisfactory	1 (1.7%)	0 (0.0%)	1 (1.6%)	Not satisfactory	0 (0.0%)	0 (0.0%)	1 (1.6%)

Data are expressed as counts and percentages
